# Construction and validation of a progression prediction model for locally advanced rectal cancer patients received neoadjuvant chemoradiotherapy followed by total mesorectal excision based on machine learning

**DOI:** 10.3389/fonc.2023.1231508

**Published:** 2024-01-24

**Authors:** Jitao Hu, Yuanyuan Sheng, Jinlong Ma, Yujie Tang, Dong Liu, Jianqing Zhang, Xudong Wei, Yang Yang, Yueping Liu, Yongqiang Zhang, Guiying Wang

**Affiliations:** ^1^ Department of General Surgery, The Fourth Hospital of Hebei Medical University, Shijiazhuang, China; ^2^ School of Information Science and Engineering, Hebei University of Science and Technology, Shijiazhuang, China; ^3^ Department of Gastrointestinal Surgery, The Third Hospital of Hebei Medical University, Shijiazhuang, China; ^4^ Department of General Surgery, The Third Hospital of Hebei Medical University, Shijiazhuang, China; ^5^ Department of Pathology, The Fourth Hospital of Hebei Medical University, Shijiazhuang, China; ^6^ The Second Hospital of Hebei Medical University, Shijiazhuang, Hebei, China

**Keywords:** deep learning, artificial intelligence, total mesorectal excision, neoadjuvant chemoradiotherapy, local advanced rectal cancer

## Abstract

**Background:**

We attempted to develop a progression prediction model for local advanced rectal cancer(LARC) patients who received preoperative neoadjuvant chemoradiotherapy(NCRT) and operative treatment to identify high-risk patients in advance.

**Methods:**

Data from 272 LARC patients who received NCRT and total mesorectal excision(TME) from 2011 to 2018 at the Fourth Hospital of Hebei Medical University were collected. Data from 161 patients with rectal cancer (each sample with one target variable (progression) and 145 characteristic variables) were included. One Hot Encoding was applied to numerically represent some characteristics. The K-Nearest Neighbor (KNN) filling method was used to determine the missing values, and SmoteTomek comprehensive sampling was used to solve the data imbalance. Eventually, data from 135 patients with 45 characteristic clinical variables were obtained. Random forest, decision tree, support vector machine (SVM), and XGBoost were used to predict whether patients with rectal cancer will exhibit progression. LASSO regression was used to further filter the variables and narrow down the list of variables using a Venn diagram. Eventually, the prediction model was constructed by multivariate logistic regression, and the performance of the model was confirmed in the validation set.

**Results:**

Eventually, data from 135 patients including 45 clinical characteristic variables were included in the study. Data were randomly divided in an 8:2 ratio into a data set and a validation set, respectively. Area Under Curve (AUC) values of 0.72 for the decision tree, 0.97 for the random forest, 0.89 for SVM, and 0.94 for XGBoost were obtained from the data set. Similar results were obtained from the validation set. Twenty-three variables were obtained from LASSO regression, and eight variables were obtained by considering the intersection of the variables obtained using the previous four machine learning methods. Furthermore, a multivariate logistic regression model was constructed using the data set; the ROC indicated its good performance. The ROC curve also verified the good predictive performance in the validation set.

**Conclusions:**

We constructed a logistic regression model with good predictive performance, which allowed us to accurately predict whether patients who received NCRT and TME will exhibit disease progression.

## 1 Introduction

Colorectal cancer is the third most common cancer worldwide and the second leading cause of cancer-related deaths. In 2020, rectal cancer accounted for 6.0% of newly diagnosed cancer cases and 3.4% of cancer deaths (1). The last decades witnessed the development of multidiscipline, individualization, and precision in treatments for rectal cancer. NCRT followed by TME has been recommended for patients diagnosed with LARC, which is correlated to lower treatment-related toxicity rate, lower local recurrence rate, and higher sphincter preserve rate (2).

However, in clinical research, the sensitivity of patients with rectal cancer to preoperative neoadjuvant therapy varies significantly, and more than half of the patients are not sensitive to neoadjuvant therapy (3, 4) and exhibit disease progression after preoperative neoadjuvant therapy and operative treatment (5). Thus, we need to accurately predict disease progression in this group of patients to target the high-risk patients for focused care and related interventions.

Current methods used for predicting the outcomes of preoperative neoadjuvant therapy include MRI imaging (6), molecular marker examination (7), blood levels (8), and the assessment of pathological and clinical characteristics (9). However, the predictions are unsatisfactory and are primarily useful for determining the effects of preoperative neoadjuvant therapy. Meanwhile, no significant progress has been made in the prediction of disease progression after preoperative neoadjuvant therapy and surgical treatment. Moreover, the routine preoperative examination of patients usually involves blood tests, such as those for neutrophil or leukocyte levels, among others. The routine preoperative examination may have better effects on predicting disease progression if multiple variables, including those available from initial tests (conducted at admission) and post-neoadjuvant examination and tests, can be used comprehensively. This would help avoid the omission of important variables and the deletion or selection of critical variables for predicting disease progression after treatment.

The significance of joint work between medcine and machine learning has been more and more recognised (10). Artificial intelligence (AI) and machine learning have been widely used to screen, diagnose, and treat patients with cancer (11). The AI risk assessment of pulmonary lymph nodes is an example. Compared to traditional statistical methods, AI techniques are more effective for handling complex data (12). Moreover, AI tools can also be built to predict the prognosis of liver cancer (13), lung cancer (14), colorectal squamous cell carcinoma (15), or breast cancer (16) in patients based on pathological images or clinicopathological characteristics. AI application represents a significant trend with potential applications in predicting the outcomes of preoperative neoadjuvant therapy and disease progression after operative treatment (17).

The prediction of disease progression after preoperative neoadjuvant therapy and operative treatment is of great significance. Moreover, previous studies had reported the prediction of the effect of preoperative neoadjuvant, such as MRI (18), circulating DNA (19), tumor microsatellite stability (20), immune cell infiltration (21), etc. However, these studies only considered a few variables, and the true magnitude of the effect needed to be clarified. Chemotherapy has become one of the most important elements in the treatment of rectal cancer (22). Preoperative neoadjuvant therapy and postoperative chemotherapy can improve the prognosis of rectal cancer patients (23). The guidelind suggest the patients recieved a total durationg of 6 months before and after operation (24), we excluded the patients didn’t recieved sufficent chemotheratpy. Here, we included information on the patients collected at admission, after neoadjuvant treatment and postoperative information such as the tumor location, colonoscopy results, imaging and postoperative pathology results. We included multiple variables in the study. We further filtered eight variables using various machine learning methods for analysis, attempting to avoid the loss of important variables. We believed this would help build a prediction model with good predictive ability, which would help predict the outcomes of neoadjuvant treatment and disease progression after operative treatment.

## 2 Materials and methods

### 2.1 Patients

A retrospective study was conducted. 272 patients diagnosed with LARC, who received NCRT and underwent TME at the 4th hospital of Hebei Medical University(Shijiazhuang, Hebei, China) were enrolled from 2011-2018 were enrolled. Data included 145 clinical variables were collected.

After considering the inclusion and exclusion criteria, data from 135 patients with rectal cancer, which included 45 clinical characteristic variables, were included in the study. All patients had undergone R0 resection after NCRT. Inclusion criteria: 1) location in the rectum, within 12 cm from the anal verge; 2) pathologically malignant and diagnosed as adenocarcinoma; 3) preoperative neoadjuvant treatment before imaging diagnosis of stage II-III disease; 4) availability of complete clinical data; 5)received standard radiotherapy: 5 days a week at 1.8 Gy per day for 5;weeks to a dose of 45 Gy, followed by a boost of 5.4 Gy, for a total dose of 50.4 Gy; 6)received complete preoperative and post-operative therapy with a duration of 6 months. Exclusion criteria: 1) Concomitant with other serious diseases, such as myocardial infarction; 2) Not receiving standard NCRT; 3) Refuse follow-up; 4)refuse to receive TME after NCRT. The patients in our study recieved standard XELOX regimen in pre-operative and post-operative chemotherapy. The research scheme was approved by the Ethics Committee of the Fourth Hospital of Hebei Medical University (Approval Code: 2023KS015).

### 2.2 Data processing

Sample data from 272 patients with rectal cancer were screened. Data from 161 patients with rectal cancer followed up for 2 years, who subsequently underwent TME after neoadjuvant therapy, were included.

One target quantity (progression) and 145 characteristic variables were selected per sample. Concurrently, One Hot Encoding was applied to certain numerical characteristics in data processing to facilitate model training. Moreover, the KNN filling method was applied for missing data attributes, whereas SmoteTomek comprehensive sampling (25–27) was used to solve the data imbalance problem to improve the classification accuracy in a few classes. Eventually, 135 patients were selected, and the final model was constructed using four machine learning methods (ten-fold cross-validation) to screen important variables for constructing the prediction model and validating it using a ROC curve.

The 145 variables were shown as followed, Gender, age, previous medical history, chief complaint, family history, smoking history, drinking history were retrospectively collected from the medical history database. As digital rectal examination, blood test, MRI, coloscopy were perfomed both before NCRT and before TME, variables from these tests were recorded twice. In digital rectal examination, the distance between toumor and anus, whether blood was observed after examination were recorded. For the tumors failed to reach through digital rectal examination, the distance was recorded through colonscopy. Variables from coloscope include: whether stenosis, edema or mucus was observed, the morphology of tumor, the status of mucosa. The level of blood tumor biomarkers inclued CEA, CA-199, CA-724, ferroprotein, β2-microglobulin were recorded. The counting of red blood cell, white blood cell, neutrophil, lymphocyte, platelet was recorded. The serum level of albumin was also recorded. Variables from MRI inculded cricumferential invasion, tumor size, clinical TNM staging, vessel invasion. For the pathological results of coloscopy biopsy, the pathological diagnosis, tumor differentiation were recorded. The exact operating method of TME and post-operative complication was also recorded. The mutation status of KRAS, NRAS, BRAF, and the expresstion status of Her-2 MLH1, MSH2, PMS2 were record through the pathological results of the operative specimens. Variable from operative specimens also included tumor size, morphology, tumor differentiation, histological grade, pathological TNM stage, blood vessel invasion, perineural invasion, tumor regression grade. The total number of post-operative The survival and progression information was collected through telephone follow-up.

### 2.3 Statistical analysis

Statistical work were completed by statistical experts (School of Information Science and Engineering, Hebei University of Science and Technology). The decision tree analysis was conducted using rpart package, random forest analysis was conducted using randomForest package, SVM was conducted using e1071 package, XGBoost was conducted using xgboost package, and LASSO regression was conducted using glmnet package. The predictive ability of the prediction models was assessed based on the AUC values of the ROC curves. P<0.05 was considered statistically significant.

## 3 Results

### 3.1 Baseline clinical characteristics

**Figure 1:**
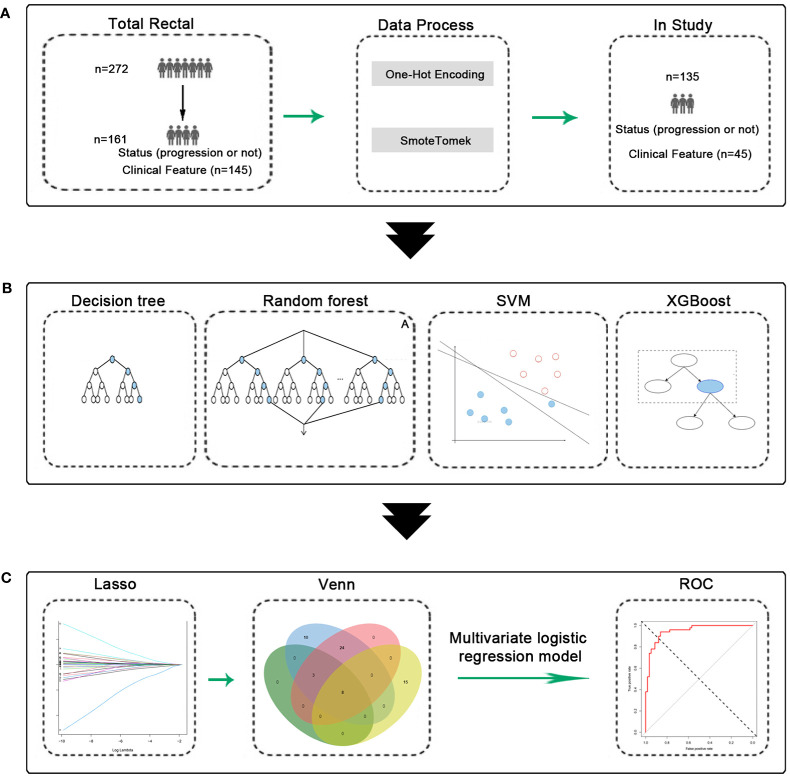
Experimental flow chart. **(A)** Data process, 135 patients were obtained. **(B)** Machine learning model construction and validation. **(C)** Construction and validation of predictive models.

We included data from 135 patients from the Fourth Hospital of Hebei Medical University who had undergone preoperative neoadjuvant therapy. Forty-five independent variables, such as gender, age, and others, were included in this study. They were randomly divided into the training and test sets in a ratio of 8:2 for subsequent analysis. There were no difference between the groups (**Supplementary Table 1**). The detailed information of 135 patients (with 45 variables) had been shown in **Supplementary Table 2**. The process was shown in **Figure 1**.

### 3.2 Machine learning model construction and validation

**Figure 2:**
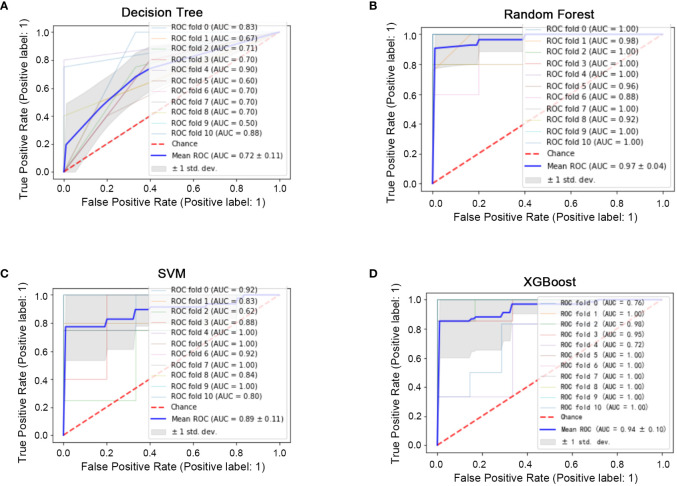
Machine learning model construction (ten-fold cross-validation) in the training set. **(A)** ROC diagram of the decision tree in the training set. **(B)** ROC diagram of the random forest in the training set. **(C)**: ROC diagram of the support vector machine in the training set. **(D)** ROC diagram of XGBoost in the training set.

**Figure 3:**
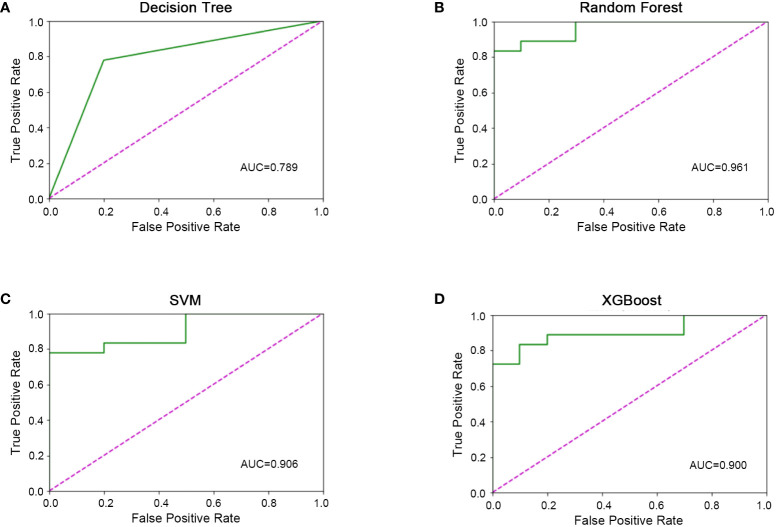
Machine learning model validation in the validation set. **(A)** ROC diagram of the decision tree in the validation set. **(B)** ROC diagram of the random forest in the validation set. **(C)** ROC diagram of support vector machine in the validation set. **D)** ROC diagram of XGBoost in the validation set.

All 45 baseline characteristics, including initial hospitalization data and preoperative data, were used to construct a model to predict whether the disease had progressed. Moreover, four machine learning methods were used in the training set to construct the models. In this model, as shown in **Figure 2**, the AUC values were 0.72 ± 0.11 for decision trees (**Figure 2A**), 0.97 ± 0.04 for random forests (**Figure 2B**), 0.89 ± 0.11 for SVM (**Figure 2C**), and 0.94 ± 0.10 for XGBoost (**Figure 2D**). To confirm the potential of the four machine learning models, we tested them in a test set and obtained similar results (**Figure 3**). Our results indicated the excellent predictive ability of the four machine learning models.

### 3.3 Predictor construction and validation

**Figure 4:**
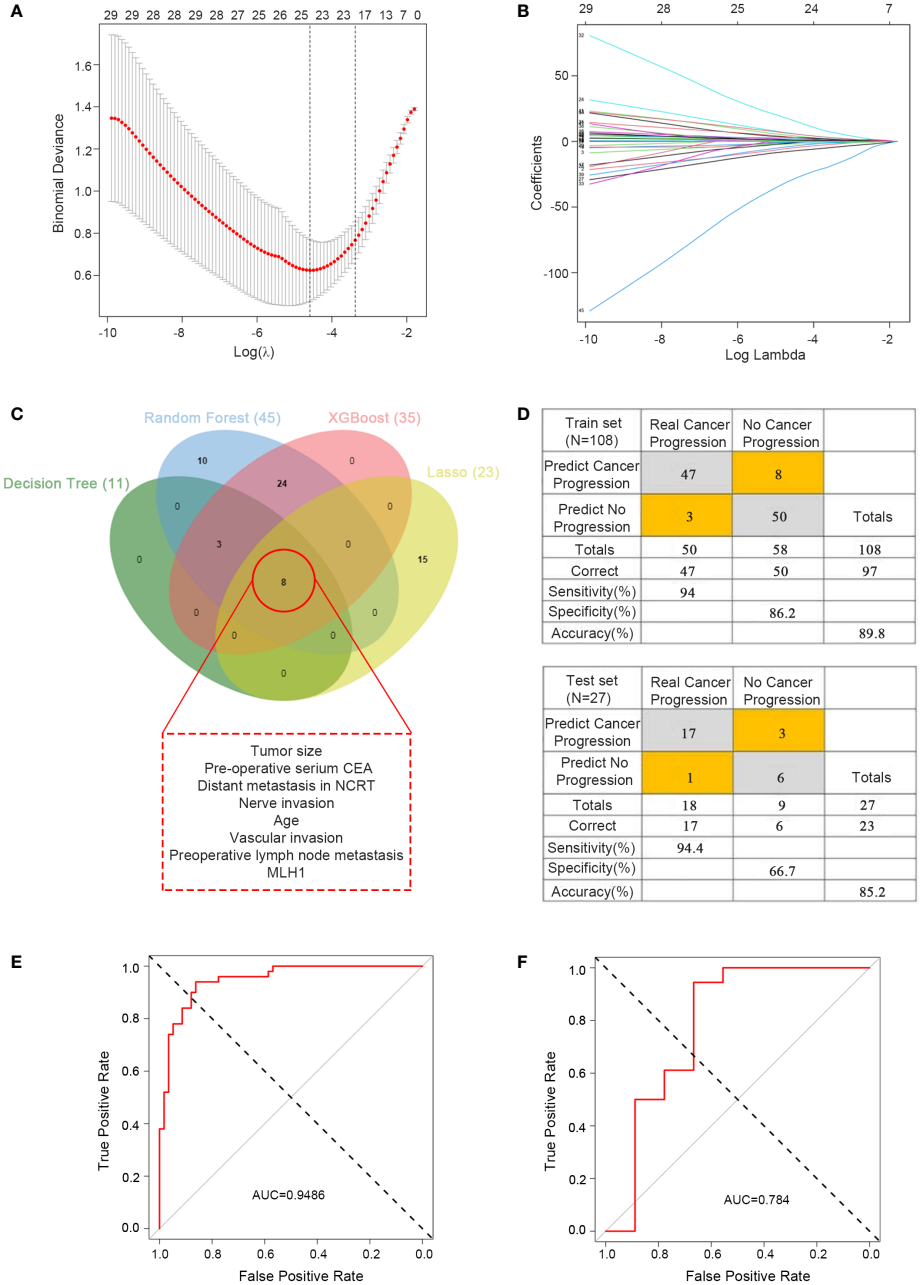
Predictor construction and validation. **(A)** Clinical characteristics of patients with rectal cancer in the LASSO model. **(B)** Selection of the tuning parameter (λ) in the LASSO model required cross-validation using the maximum criteria. **(C)** Venn diagram of the outcomes of the four machine learning methods for filtering variables. **(D)** Confusion matrix of binary outcomes after logistic regression for predicting patient progression in rectal cancer, the predictor for the train set (upper) and test set (lower). **(E)** ROC curves for predicting disease progression in patients with rectal cancer undergoing preoperative neoadjuvant therapy and after surgical treatment to distinguish whether progression; the training set. **(F)** ROC curves for predicting disease progression in patients with rectal cancer undergoing preoperative neoadjuvant therapy and after surgical treatment to distinguish whether progression; the test set.

The predictive ability of the four machine learning models werre good; however, with so many variables, it was not very convenient for practical applications. To further reduce the number of variables, we performed LASSO regression analysis, which yielded 23 variables identified (**Figures 4A, B**) and eight critical variables (tumor size, pre-operative serium CEA, distant metastasis in NCRT, nerve invasion, age, vascular invasion, preoperative lymph node metastasis, MLH1) were identified using a Venn diagram by four methods (**Figure 4C**). The MLH1-status was assessed by immunohistochemistry(IHC). These eight variables were subsequently used for multivariate logistics regression to construct a diagnostic prediction model with a discriminant optimal cutoff value of 0.314, suggesting that patients with scores <0.314 could be considered progression-free and patients with scores >0.314 could be considered to exhibit progression. In the training set, ROC analysis revealed a sensitivity of 94% and a specificity of 86.2% for the differentiation between progression and non-progression (**Figure 4D** upper), with an AUC value of 0.9486 (**Figure 4E**). Similar results were obtained in the validation set, with a sensitivity of 94.4%, a specificity of 66.7% (**Figure 4D** down), and an AUC value of 0.784 (**Figure 4F**).

## 4 Disscussion

Preoperative neoadjuvant chemoradiotherapy was an important part of rectal cancer treatment (28), and many previous studies had reported prediction models for the response of rectal cancer to preoperative neoadjuvant therapy (18, 29, 30). However, cases of progression after preoperative neoadjuvant therapy could not be ignored. To rule out the differences caused by chemotherapy, we ultimately included patients who had undergone sufficient chemotherapy in the study to minimize the bias caused by individual chemotherapy as much as possible. In our study, we obtained eight critical variables using four machine learning methods to construct a prediction model for progression after preoperative neoadjuvant therapy, which can reasonably predict the disease progression of patients. This can help improve the focus and increase the frequency of reviews in such cases. Additionally, once signs of progression were detected, the treatment plan could be altered immediately. This can help avoid delays in treatment and improve patient prognosis.

Machine learning have been widely applied in clinical decision-making (10). For example, machine learning had been previously applied to readmission after elective laparoscopic colorectal surgery (31). Tumor burden before and after NCRT are depcited through cTNM before NCRT stage and ypTNM respectively. However, the joint effects of pre and after NCRT stage is not well-studied. A system that is able to integret multiple information may better predict the prognosis of patients (32).

In our study, we included more than 100 variables and tested comprehensive data to avoid missing variables that can influence disease progression. To our knowledge, the variables included in our study were numerous.

We initially constructed four machine learning models. Even though the AUC values were high, they all showed good predictive functions in the training set and test set, but the value of 0.9 did not meet our requirements. Hence, we further screened the variables by LASSO regression and then using Venn diagram to further screen variables. We eventually selected eight important variables. The AUC value of the final prediction model was considerably high at 0.9486, indicating the excellent function of our model. The applicability of these eight variables was high because they were mandatory examinations or tests for patients who require hospitalization.

Feature selection plays a crucial role in the field of machine learning, as it can select the most informative features from raw data, improve model performance, reduce overfitting, and accelerate model training and prediction speed. In large-scale datasets and high-dimensional data, feature selection is particularly important because unnecessary features increase computational complexity and introduce redundant information (33, 34). When selecting univariate and multivariate regression analysis, we need to have an adequate sample size, with a positive sample size at least 10 times the number of variables. The more the better, in order to meet the meaningful results. In addition, we believed that the feature selectionn of univariate and multivariate regression carries subjectivity (subjective selection of p-value), while the feature selection of machine learning relies on computation and is more observable. In sum, univariate and multivariate regression focus more on analyzing the impact of independent variables on outcomes, while feature selection is a part of machine learning.

In the features selection for model construction, SVM was excluded from the analysis. The machine learning of this study were based on the sklearn framework. Decision trees, random forests, and XGBoost were all based on the important features of tree models, so the important features can be obtained from the model. However, SVM did not have important features in the algorithm, so important features were not be obtained. Therefore, in the selection of the variables, SVM was excluded from the analysis.

Currently, nearly all the prediction models for rectal cancer patients undergoing neoadjuvant treatment are used to predict tumor response to identify sensitive patients. Here, we aimed to predict tumor progression after neoadjuvant treatment, to identify high-risk patients. Thus, we pay more attention to these high-risk patients, benefiting for early detection and early treatment. CEA, known as a biomarker in colorectal cancer, had been reported to be associated with pathological complete remission after neoadjuvant treatment for rectal cancer, and tumor size and preoperative CEA are related to tumor downstaging (35). So tumor size and preoperative CEA had the potential to predict tumor progression. Similarly, distant metastasis, nerve invasion, age, vascular invasion, and preoperative lymph node metastasis are all related to tumor prognosis (36, 37), revealing the potential to predict tumor progression. These 7 variables are routine preoperative examination items, indicating that our model had good generality.

However, the time point at which progression occurred and was concentrated remains unascertained, which was also a limitation of this study. In our future studies, we will focus on this aspect of the research topic to determine the period in which the disease is more prone to progression. In addition, This study was a single center retrospective study, and the model constructed lacked external data validation. There was also a selection bias in this study due to the missing cases in the study. In the future, we would collaborate with other centers to further increase the sample size, validate and optimize the model constructed in this study. This will help reduce the frequency at which reviews are conducted and help focus on reviews during critical periods. This is also conducive to adjustments in treatment plans based on the availability of medical resources. In conclusion, we have constructed a model with good predictive function and wide applicability, which can help improve the focus on critical patients and their prognosis.

## 5 Conclusion

We constructed a logistic regression model with good predictive performance, which allowed us to accurately predict whether patients who received NCRT (sufficent standard XELOX regimen) and TME will exhibit disease progression.

## Data availability statement

The original contributions presented in the study are included in the article/**Supplementary Material**. Further inquiries can be directed to the corresponding authors.

## Ethics statement

The studies involving humans were approved by The research scheme was approved by the Ethics Committee of the Fourth Hospital of Hebei Medical University (Approval Code: 2023KS015). The studies were conducted in accordance with the local legislation and institutional requirements. Written informed consent for participation was not required from the participants or the participants’ legal guardians/next of kin because Our research was a retrospective study and did not require the written informed consent.

## Author contributions

All authors contributed to the research conception and design. Conceptualization, GW, YZ and YL; methodology, YS, JH; software, YS, YZ, GW; validation, JZ; formal analysis, YT; investigation, JH, DL; resources, GW; data curation, JM; writing—original draft preparation, YY; writing—review and editing, YL; visualization, XW; supervision, JH; project administration, JH; funding acquisition, GW. All authors have read and agreed to the published version of the manuscript.
